# Death by Hanging

**Published:** 1851-11

**Authors:** 


*Death by Hanging* — Post Mortem examination, made twenty-four hours after death, upon the two negroes, Henry and Moses, executed at Nashville on the 21st February, 1851. By R. M. PORTER, M. D., of Nashville, Tenn.

The brain was congested in both cases. The vessels supplied with blood of a dark venous hue; those supplied by the vertebral arteries preserved their natural aspect, the blood retaining its scarlet arterial color. The two kinds of blood were very distinctly seen at the termination of the basilar artery in the circle of Willis. The sinuses of the dura mater were full to distension.

In both subjects, there was considerable effusion of serum into the arachnoid cavities, sub-arachnoid cellular tissue and ventricles — to such an amount as probably to have caused serous apoplexy. There was no extravasation of blood in any part, nor any appreciable lesion of the cerebral substance. There was no fracture, nor serous laxation of the cervical vertebræ.

In both cases the lungs were very much engorged, and had the dark purplish tinge of *splenization.* By pressing any portion of the lungs between the fingers, the pulmonary tissues were made to assume their natural color. The blood was fluid in every part of the body: venous congestion was very marked; the left side of the heart was empty; the right side was full.

The mark of the rope around the neck was left, looking as if the skin had been seared with a hot iron, and there was a contusion of the superficial layer of muscles corresponding with the impression on the surface. In Henry, much the heavier body of the two, the sterno-cleido mastoid muscle was nearly severed, leaving only a few fibres on the right side opposite that on which the hangman's knot was placed.

In the thorax of Moses, an abscess was found nearly ready to burst; it contained three or four ounces of pus. This being evacuated, it was ascertained that the fourth and fifth dorsal vertebræ were carious and the intervertebral substance was nearly destroyed. All the lumbar vertebræ were carious and an abscess was formed on the right side containing about half a gallon of purulent matter. On the left side was a smaller abscess. There was also a small abscess on the right elbow joint. Yet there was nothing in the external appearance or configuration to indicate that he was not a healthy negro. There was extensive pleuritic adhesion in both cases.

Inspection of the bodies a few hours after the execution, showed that there had been an emission of semen from both, but no relaxation of the sphincter of the anus or bladder. There was some tumefaction of the face and lividity of the lips; the eyes wore their natural appearance. The jaws were firmly locked; the tongue protruding slightly and deeply indented by the teeth; *rigor mortis* had not taken place in the limbs. The bodies were still warm.

The following extract from the London Quarterly Review may not be without interest in connection with this subject:

"An immense number of persons recovered from insensibility (produced by hanging) have recorded their sensations, and agree in the report that an easier end could not be desired. An acquaintance of Lord Bacon, who meant to hang himself partially, lost his footing, and was cut down at the last extremity, having nearly paid for his curiosity with his life. He declared that he felt no pain, and his only sensation was of fire before his eyes, which changed first to black and then to sky-blue. These colors are even a source of pleasure. A captain Montagnac, who was hanged in France during the religious wars, and rescued from the gibbet at the intercession of Viscount Turenne, complained that having lost all the pain in an instant, he had been taken from a light of which the charm defied description. A criminal who escaped by the breaking of the cord, said that after a second of suffering, a fire appeared and across it a most beautiful avenue of trees. Henry IV, of France, sent his physician to question him, and when mention was made of a pardon, the man answered that it was not worth the asking. The uniformity of description renders it useless to multiply instances. All agree that the uneasiness is quite momentary, that a pleasurable feeling immediately succeeds, that colors of various hue start up before the sight, and that these having been gazed on for a trivial space, the rest is oblivion. The mind, averted from the reality of its situation, is engaged in scenes the most remote from that which fills the eye of the spectator — the vile rabble, the hideous gallows, and the struggling form that swings in the wind."

Nashville, April 17th, 1851. — *Nashville Jour. of Med. and Surg.*

